# Grain Structure Control of Additively Manufactured Metallic Materials

**DOI:** 10.3390/ma10111260

**Published:** 2017-11-02

**Authors:** Fuyao Yan, Wei Xiong, Eric J. Faierson

**Affiliations:** 1 Department of Materials Science and Engineering, Northwestern University, Evanston, IL 60201, USA; 2 Department of Mechanical Engineering and Materials Science, University of Pittsburgh, Pittsburgh, PA 15261, USA; 3 Quad City Manufacturing Laboratory-Western Illinois University, Rock Island, IL 61201, USA; efaierson@qcml.org

**Keywords:** metal additive manufacturing, grain morphology, grain size, post-processing heat treatment

## Abstract

Grain structure control is challenging for metal additive manufacturing (AM). Grain structure optimization requires the control of grain morphology with grain size refinement, which can improve the mechanical properties of additive manufactured components. This work summarizes methods to promote fine equiaxed grains in both the additive manufacturing process and subsequent heat treatment. Influences of temperature gradient, solidification velocity and alloy composition on grain morphology are discussed. Equiaxed solidification is greatly promoted by introducing a high density of heterogeneous nucleation sites via powder rate control in the direct energy deposition (DED) technique or powder surface treatment for powder-bed techniques. Grain growth/coarsening during post-processing heat treatment can be restricted by presence of nano-scale oxide particles formed in-situ during AM. Grain refinement of martensitic steels can also be achieved by cyclic austenitizing in post-processing heat treatment. Evidently, new alloy powder design is another sustainable method enhancing the capability of AM for high-performance components with desirable microstructures.

## 1. Introduction

In recent years, metal additive manufacturing (AM) has become a new revolutionary technology for industrial manufacturing systems. It is a process in which a local high-power heat source melts the newly deposited material and a small volume of underlying pre-deposited material, and lets them solidify into one solid piece as the heat source moves away. The heat source moves in accordance to path planning software, in which the degree of user control can vary to a large degree from system to system. The material is built up in a layer-by-layer manner, which allows for the creation of parts with complex shapes and internal features that cannot be produced by conventional subtractive manufacturing methods.

**Figure 1:**
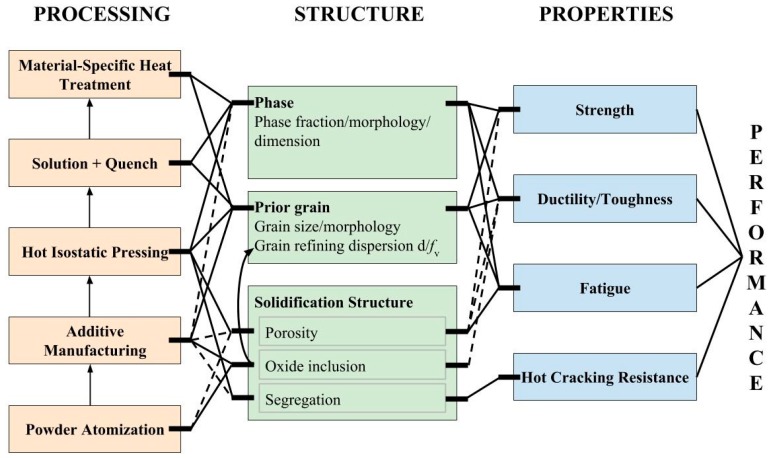
Systems design chart outlining the major process-structure-property-performance relationship for general metallic materials produced by powder-based additive manufacturing. Dash lines imply that the effects can be eliminated by following processing steps.

The multiple melting-solidification cycles during AM processes directly result in complex microstructures varying spatially within the build. Typical macro-micro/structural features include material discontinuities, highly textured columnar grains, complex phases, and compositional variations [1]. People have made great efforts to understand the process-structure-property-performance (PSPP) relations in AM [2,3,4,5] with the intent to produce desirable microstructural features, and thus to achieve comparable or even superior mechanical properties to conventionally manufactured materials. A general systems design chart, shown in Figure 1, outlines the major PSPP relationships for powder-based AM alloys. Proper designs on different processing steps such as powder atomization, laser/electron beam heating, and related post-processing heat treatments can greatly optimize microstructures and enhance mechanical properties. It is pointed out that AM process directly influences all the listed microstructural features. However, except for grain structure and inclusion distribution, most of these features inherited from the building processes can be eliminated by post heat-treating. Therefore, some of these processing-structure linkages can be understood and described using conventional metallurgical models. Since the evolution of grain structure is greatly influenced by all processes performed, as well as by inclusions serving as Zener-pinning particles, it is critical to control the grain structure by tuning AM process parameters and post heat-treatment conditions.

Grain structure control and optimization is commonly used to create equiaxed fine grains in order to provide both strength and ductility enhancement. Unfortunately, work hardening to promote recrystallization is not applicable in AM, so it is challenging to establish a feasible method to refine grain structure for strengthening purposes. In this work, grain structure optimization in-situ and with post process will be discussed, which then assist on AM alloy and processing designs.

## 2. Grain Morphology Control

### 2.1. Via AM Processes

**Figure 2:**
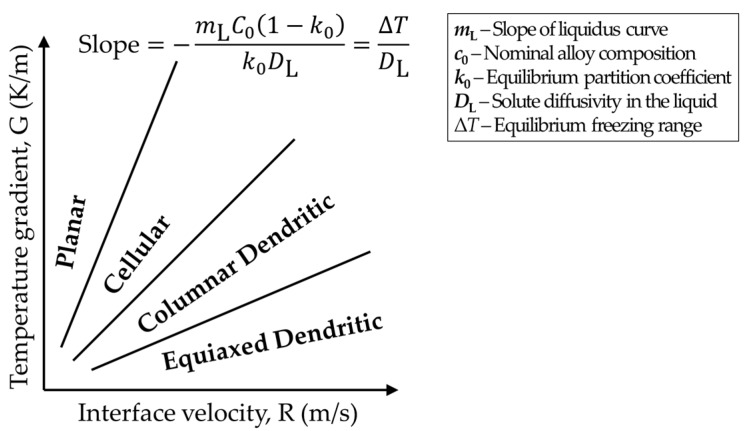
Schematic representation of the combined effect of thermal gradient and solidification velocity on solidification microstructure [14].

The as-built alloys usually exhibit unique grain morphologies. Typical as-solidified grain morphology, which has been widely observed in various materials produced by different AM methods, is highly textured large columnar grains spreading over several layers along the build direction as a result of epitaxial growth [6,7,8,9]. Additionally, fine equiaxed grains near the melt pool surface are also frequently observed [9,10]. According to the theories of alloy solidification, due to constitutional supercooling, the solid/liquid interface morphology can be planar, cellular or dendritic, depending on the solidification condition and the material composition [11]. Constitutional supercooling occurs with solute redistribution which causes the liquid at the solidification front to be cooler than the liquidus temperature. For a specific alloy, the degree of constitutional supercooling is determined by the ratio of thermal gradient (G=|∇T|) and the kinetics of mass transfer (i.e., solidification rate velocity, R=(1/G)(∂T/∂t)). The combined effect of *G* and *R* on morphology transitions in alloy solidification is schematically shown in Figure 2. The increase of *G*/*R* results in solidification mode change and therefore morphology change in the order: equiaxed dendritic, columnar dendritic, cellular and planar. It has been reported that typical *G*/*R* for IN718 produced by selective laser melting (SLM) is calculated as 20~200 K·s/mm^2^, which is far below the lower limit (7000 K·s/mm^2^) for planar solidification to occur [12]. It is generally found that *G*/*R* near the bottom of the melt pool fall in the region for columnar-dendritic solidification, whereas the equiaxed solidification condition may be satisfied near the melt pool surface where the thermal gradient is lower than that at the bottom of the pool. In order to achieve equiaxed solidification, a lot of work has been done to calibrate the columnar-to-equiaxed transition (CET) regions for various materials systems [2,7]. For AM processes, the adjustment of *G* and *R* is achieved by the manipulation of AM process parameters (i.e., heat source power, scan speed, scan strategy, spot size, preheating temperature, etc.), with the aid of heat transfer and fluid flow models. For parts with complex geometries, different locations may respond differently and experience different thermal histories under the same AM conditions [13]. To ensure desirable *G*-*R*, special tuning of AM process parameters is needed in accordance with location-specific thermal simulations.

**Figure 3:**
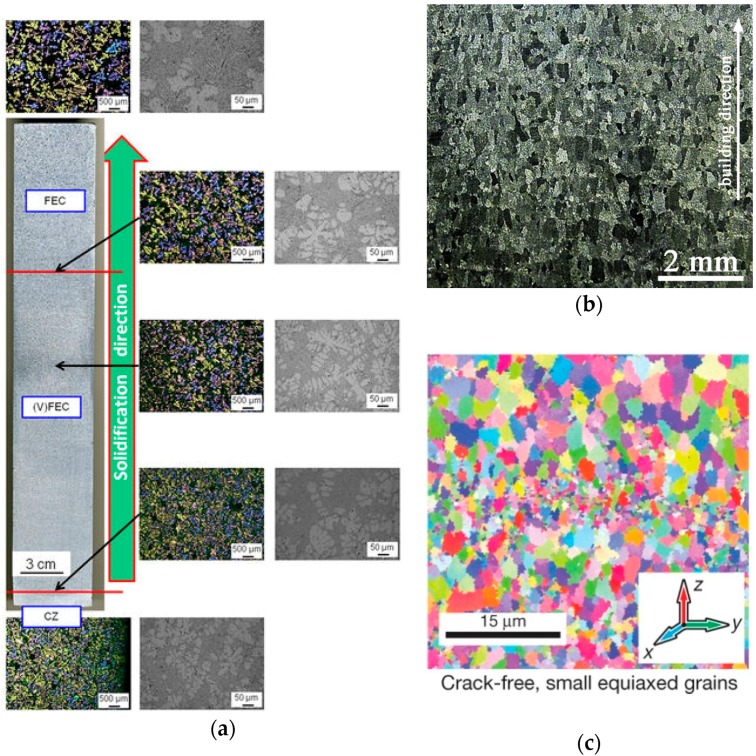
(**a**) Microstructure of the directionally solidified Al-10Si alloy with inoculation of Nb-based heterogeneous nuclei. CZ: chilled zone, (V) FEC: (very) fine equiaxed crystals. Adapted from Bolzoni [16]; (**b**) Optical micrographs showing fine near-equiaxed grains on the transverse cross section of the thick-plate titanium component produced by direct energy deposition (DED) at laser power 6 kW, beam diameter 6 mm, beam scan rate 1000 mm/min and mass deposition rate 50 g/min. Adapted from Wang [17]; (**c**) Fine equiaxed grains of selective laser melting (SLM)-processed aluminum alloy in the as-built condition produced with modified powders. Adapted from Martin [18].

As mentioned above, one solution to promoting equiaxed solidification is to create a long and constitutionally supercooled region by decreasing *G*/*R*, if the temperature gradient (*G*) is not strongly dependent on the gradient direction. During equiaxed solidification, equiaxed grains are the hottest part in the melt, and the heat flow direction is the same as the grain growth direction, whereas during dendritic solidification, columnar dendrites are the coolest part as they grow in the opposite direction to the heat extraction. It is therefore possible to induce equiaxed solidification near the top of the melt pool through surface cooling [11], which can be achieved in the AM process as the cool inert gas flows into the chamber across the melt pool. If the melt pool contains a sufficient number of inoculants, heterogeneous nucleation of new grains ahead of the advancing solid/liquid interface is greatly promoted. The number density of inoculants may alter the CET, as indicated by Equation (1) [15],

(1) $$G<0.617N_{0}{}^{1/3}\Delta T_{c}\left(1-\frac{\Delta T_{n}{}^{3}}{\Delta T_{c}{}^{3}}\right)\Delta T_{c}$$

where *N*_0_ represents nucleant density (1/m^3^), Δ*T*_c_ represents solute undercooling of dendrite tip (K) and Δ*T*_n_ represents nucleation undercooling (K). Equation (1) addresses the criterion for equiaxed growth to occur as the volume fraction of equiaxed grains greater than 0.49 when the columnar front passes. It can be further derived that as more inoculants are injected into the melt, more nucleation sites are provided, and the equiaxed region in the *G*-*R* map is extended. Bolzoni et al. [16] produced non-uniform and fine equiaxed dendritic structure in the directional solidification of Al-10Si alloy by inoculating Al-2Nb-*x*B compounds, as shown in Figure 3a. For powder AM, the partially melted or the residual metallic powders can serve as heterogeneous nucleation sites, especially near the top of the melt pool. Wang et al. [17] investigated the influence of deposition rate (or powder flow rate) on the grain morphology evolution in a titanium alloy built by direct energy deposition (DED). They pointed out that high specific deposition rate in DED results in insufficient powder melting and therefore enormous heterogeneous nucleation sites not only at the melt pool surface, but within the melt pool, restraining epitaxial growth at the bottom of the melt pool. The high specific mass deposition rate also reduces the laser penetration depth, so that the equiaxed grains in the previous layer are preserved, as shown in Figure 3b. However, over-flowing powders in DED may bring in high risk in producing lack-of-fusion porosity filled with un-melted powders. For powder-bed AM processes, where the amount of powders within a melt pool cannot be easily adjusted, Martin et al. [18] coated 7075 and 6061 aluminum powders with 1 vol % hydrogen-stabilized zirconium particles. The nano-particles are first dragged into the melt pool and form nano-Al_3_Zr, which serve as nucleants ahead of solidification front to promote equiaxed grain growth, as shown in Figure 3c.

### 2.2. Via Post Heat-Treatments

**Figure 4:**
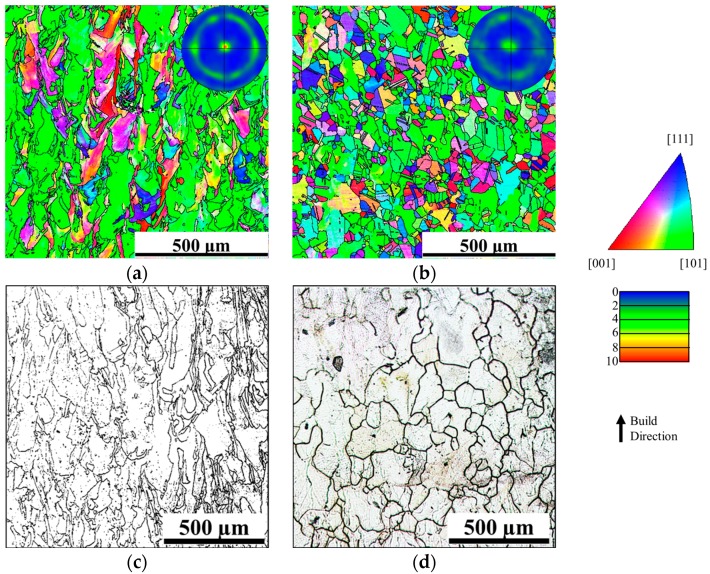
Evolution of grain structure along the build direction of SLM 316L during post-processing heat treatment. (**a**) Inverse pole figure (IPF) color map showing the as-deposited microstructure, with the subset for [110] build direction pole figure for the austenite phase; (**b**) IPF color map showing the microstructure after heat treatment at 1200 °C for 30 min, with the subset for [110] build direction pole figure for the austenite phase; (**c**) Optical micrograph showing grain structure after heat treatment at 800 °C for 1 h; (**d**) Optical micrograph showing grain structure after heat treatment at 1100 °C for 1 h.

As a remedy for porosity in the as-built alloys, hot isostatic pressing (HIP) is always performed at high temperatures and high pressures for a couple of hours after AM processing in order to close and eliminate internal voids [19]. HIP can be regarded as a homogenization process performed in the single-phase field at a very high temperature. Therefore, the residual stress induced by repeated heating and cooling during AM process, can be released during HIP in the form of recrystallization of equiaxed grains. Figure 4 shows the grain structure evolution of 316L stainless steel produced by SLM during heat treatment. Small equiaxed grains appear after 30-min holding at 1200 °C as in Figure 4b, and partially take place of columnar grains which are textured along <110> directions in the as-built condition as in Figure 4a. Recrystallization is also evident as the texture disappears indicated by the <110> pole figures along the build direction. The recrystallization temperature is the temperature at which recrystallization reaches 50% completion within 1 hour. Therefore, the recrystallization temperature for AM materials can be experimentally estimated through a 1-h heat treatment between 800 °C and 1200 °C with a 100 °C interval. Columnar grain structure is still present at temperatures below 1100 °C, as indicated in Figure 4c. The formation of equiaxed grains start to be observed at 1100 °C, as in Figure 4d. As a result, heat treating temperature for SLM 316L should be above 1100 °C to trigger sufficient recrystallization. The recrystallization process can be accelerated at a higher temperature, as in Figure 4b, but the temperature also needs to be below the δ-formation temperature to stay in one phase field. The feasibility of recrystallization during post-processing heat treatment is greatly dependent on the amount of residual stress stored in the as-built materials, which varies with AM process parameters and material types. For example, comparing with laser melting, electron beam melting (EBM) processes can introduce slower cooling rate due to potentially higher pre-heating temperatures on building substrate and larger hatch spacing, less residual stress may be stored in the as-built materials to induce recrystallization [20]. For SLM Ti-6Al-4V alloy, the recrystallization response to heat treatment is not as obvious as that in SLM 316L. After β-annealing, long columnar β grains are found to become large equiaxed grains, with length unchanged but width increased, implying extensive grain growth [21]. A possible explanation may be insufficient stored energy in the as-built alloy to trigger recrystallization, since the microstructural defects that contribute to the stored energy annihilate rapidly due to fast diffusivity of atoms in titanium alloys.

Considering the complicated distribution of residual stress in the as-built component, which is dependent on the geometry of the part, materials properties, and AM processing conditions [22], it is common for non-uniform recrystallization and thus grain distribution over the entire part. Since the stored energy in the AM part cannot be matched with those in mechanically-worked materials, it is reasonable to expect slight or even no recrystallization phenomenon in AM materials.

## 3. Grain Size Optimization

Grain size affects the mechanical properties of a material and is a result of both the AM process and a series of post-processing heat treatments. It is important to generate fine grain structures in the as-solidified condition to ensure good mechanical properties, and it is also critical to inhibit grain growth in the subsequent heat treating steps.

### 3.1. Via AM Processes

**Figure 5:**
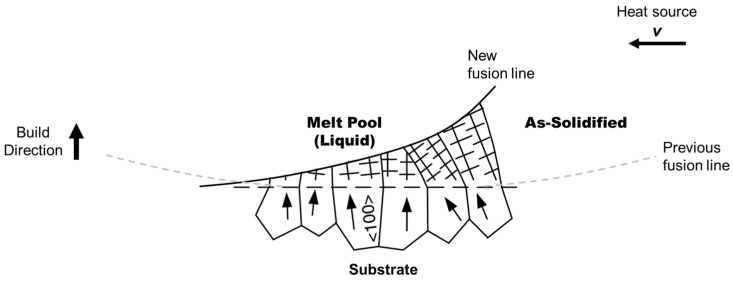
Schematic illustration of competitive epitaxial grain growth in the melt pool during solidification as the heat source moves away. The arrows in the grains of the substrate indicate the easy growth direction (i.e., <100> in fcc and bcc metals) [11].

The re-melting of the previous layer during AM generally induces heterogeneous nucleation at the melt pool boundary and epitaxial grain growth with cellular or dendritic solidification front. Therefore, grain size of the substrate determines the transverse columnar grain size. As a result of competitive epitaxial grain growth, only grains with their easy growth direction (e.g., <100> for fcc and bcc metals, and <1010> for hcp metals) parallel to the direction of the maximum temperature gradient grow easily, and as a result crowd out other grains whose easy growth direction deviate significantly from the maximum temperature gradient [11], as schematically shown in Figure 5.

If the epitaxial growth of columnar grains is restrained by the formation of equiaxed grains near the surface of the melt pool, and the equiaxed grain depth within the melt pool is greater than the penetration depth during re-melting, equiaxed grain size then dominates the average transverse grain size. For AM processes, equiaxed grain size is greatly determined by the number density of heterogeneous nucleation sites, which is usually easily controlled during the DED process where powder flow rate is one of the user settings [17].

**Figure 6:**
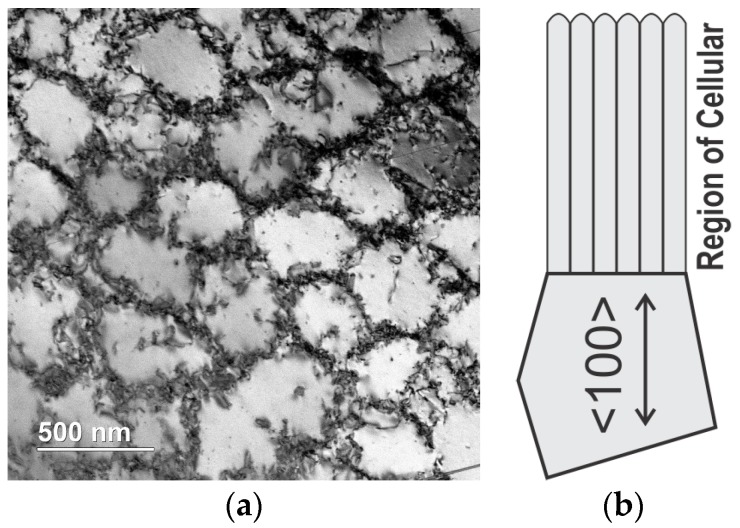
(**a**) Cellular structures in SLM 316L, with arrays of dislocations piling up at cell boundaries. Build direction points out of the paper; (**b**) Schematic illustration of grain boundaries and cell boundaries [11].

Cooling rate, in the form of G×R, has been frequently discussed to achieve finer microstructural features, such as finer cell/dendrite spacing, and therefore enhance yield strength of as-built materials compared with wrought materials [23]. Since cell boundaries are generally low-angle boundaries, i.e., arrays of dislocations shown in Figure 6a, the cells can be easily eliminated by subsequent heat treatment. What really influences the yield strength of the end-part is the size of the grains, which appear as a cluster of cells/dendrites in the as-built structure, as schematically illustrated by Figure 6b. If epitaxial grain growth is dominant, the grain size of the substrate therefore determines the final transverse grain size in the as-built materials. If equiaxed solidification occurs near the top of the melt pool, grains at the bottom of the melt pool in the following layer then epitaxially grow from the equiaxed grains in the pre-deposited layer, and inherit the equiaxed grain size which is controlled by the cooling rate.

### 3.2. Via Post Heat-Treating Processess

**Figure 7:**
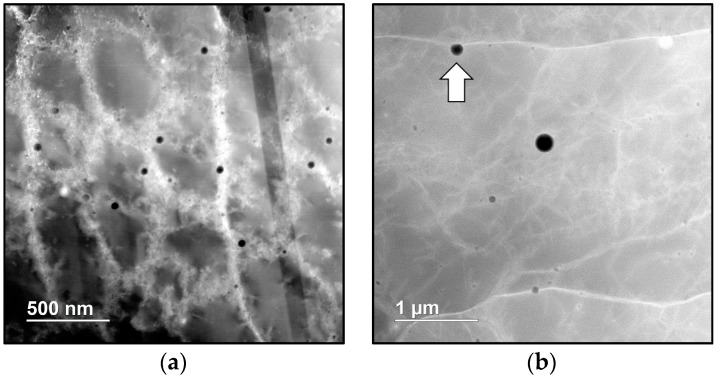
(**a**) Z-contrast scanning transmission electron micrograph showing nano-scale MnO-SiO_2_ rhodonite particles distributed along cell boundaries in the as-deposited SLM 316L.; (**b**) Microstructures of SLM 316L heat-treated at 1200 °C for 30 min. The arrow shows a MnO-SiO_2_ rhodonite particle acting as an effective Zener-pinning particle that pins the grain boundary.

Grain growth and coarsening can occur during the post-processing heat treatment, and are driven by a reduction in interfacial energy. The presence of Zener-pinning particles, which are usually sized around 100–200 nm, can effectively restrain grain growth to a much lower rate [24]. One type of effective Zener-pinning particles are MC (M = Ti, V, Nb) carbides in steels, which are deliberately alloyed and form upon heat treatment in the MC+γ phase field [25]. In recent studies, nano-scale Si-rich oxides are discovered in the as-deposited SLM 316L stainless steel [26,27], as shown in Figure 7a. They are pointed out to in-situ provide considerable strengthening to the as-built materials, as in oxide dispersion strengthening (ODS) steels, and also to serve as Zener-pinning particles, as shown in Figure 7b, which inhibit grain growth during high-temperature heat treatment. Deoxidation reactions in steels during solidification have been studied extensively in casting and welding societies [28,29,30]. With conventional manufacturing methods, oxide inclusions are of micron-scale, and are taken as defects in materials where cracks are typically initiated. Due to extremely high cooling rates during AM processes, the growth of oxides is highly suppressed down to a sub-micron scale. Thus, by taking advantage of residual oxygen from powders and the chamber to form nano-scale oxide particles during AM processes, deliberate addition of grain refiners may no longer be necessary.

**Figure 8:**
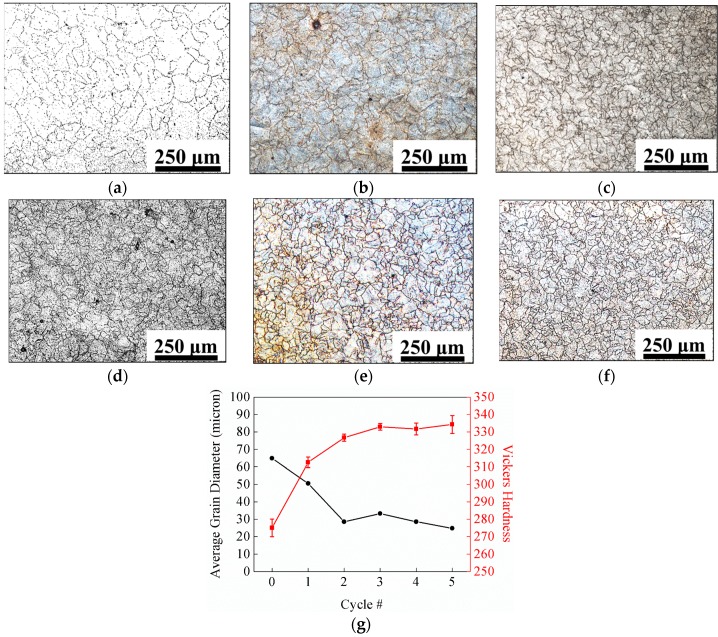
Grain structure of DED PH48S after cyclic austenitizing. (**a**–**g**) Grain morphologies: (**a**) as-homogenized (0 cycle); (**b**) 1 cycle; (**c**) 2 cycles; (**d**) 3 cycles; (**e**) 4 cycles and (**f**) 5 cycles; (**g**) Grain size and microhardness vs. cycles of austenitizing. Adapted from Yan [31].

Another traditional treatment to achieve grain refinement is to apply large deformation to the materials and then heat treat the materials to trigger recrystallization, so that large deformed grains can be replaced by fine equiaxed grains. Since AM is a near net-shaping process, it is not suitable to apply external mechanical work on AM builds. Therefore, post processing that can initiate recrystallization without changing the shape of the objects needs to be employed to refine the grains. For martensitic steels, one possible way to engage recrystallization for grain refinement is to perform a cyclic austenitizing process, which involves cycles of short duration and low-temperature austenitizing followed by quenching to form martensite. The martensitic transformation can generate a high density of dislocations, which can drive recrystallization during austenitizing. An example is presented in Figure 8, which demonstrates the effectiveness of grain refinement by cyclic austenitizing on PH48S stainless maraging steel produced by DED.

## 4. Perspectives on the Design of New Materials for AM

Currently, there are only a few types of alloys commercially available for AM. Considering the unique melting-solidification cycles during AM and the limitations in achieving grain refinement by large deformation of the as-built alloys, it is necessary to optimize the existing AM powder materials or develop new powder materials suitable for AM, so that desirable grain structure can be easily achieved with good compatibility to AM thermal histories, such as low susceptibility to hot cracking.

To promote equiaxed grain growth during AM, sufficient heterogeneous nucleation sites and an appropriate temperature field are two pre-requisites. The creation of heterogeneous nucleation sites is primarily achieved by alloy powder design (such as powder composition) and process optimization (such as powder flow rate in DED). One of the design concepts is the formation of sub-micron intermetallics during solidification. Such intermetallics need to be deliberately chosen to have the smallest mismatch with the matrix, so that intermetallics can also provide considerable modulus strengthening. Constitutional supercooling has been found to promote heterogeneous nucleation of new crystals and the formation of an equiaxed zone during solidification [32]. For a constant temperature gradient, a greater constitutional supercooling inclines to promote equiaxed solidification, which requires a higher freezing range of the alloy [1]. Freezing range *P* is termed as the temperature difference between liquidus and solidus, which can be calculated by *P* = *m*_L_*c*_o_(*k* − 1)/*k* (*m*_L_—the slope of liquidus curve, *c*_o_—nominal alloy concentration, *k*—equilibrium partition coefficient). However, a large freezing range may raise the tendency toward hot cracking during solidification, as a result of failure in liquid feeding into inter-dendritic regions. Qian et al. [33] linearly related the reciprocal of the growth restriction factor *Q* = *m*_L_*c*_o_(*k* − 1) with the average grain size during solidification, and a large *Q* leads to a fine grain size. This method can predict the actual grain size if *Q* is given for a specific alloy system. *Q* can be calculated with the aid of thermodynamic databases, by using commercial software, such as Thermo-Calc.

In terms of grain growth restriction, oxide particle size needs to be well constrained to the sub-micron range during the AM process. Since oxides typically have high solvus temperature, it is impossible to dissolve oxides and to form more highly distributed nano-particles. If the solvus temperature of the oxide is reduced, the oxide may solidify at a lower temperature and there will be less time for the oxide to grow during rapid solidification.

## 5. Conclusions

In this work, grain structure optimization has been discussed with respect to the AM process and post-processing heat treatments. For grain morphology, methods to promote equiaxed grains during solidification and post-processing heat treatment are discussed. Grain morphology of as-deposited materials is mainly controlled by CET, which requires the manipulation of *G* and *R* that are determined by AM process parameters. The introduction of enormous heterogeneous nucleation sites via powder rate control in DED or powder surface treatment for powder-bed techniques can effectively induce equiaxed grains during solidification. Recrystallization during high-temperature homogenization has been observed in steels and nickel superalloys processed by SLM, but the phenomenon is slight, non-uniform and un-controllable. Grain size of the as-deposited materials can be greatly influenced by the number density of nucleation sites. Cyclic austenitizing is an effective post heat-treating method for grain refinement of AM martensitic steels. For alloys that do not exhibit martensitic transformation during fast cooling, grain growth may be restrained by in-situ formed nano-scale oxide particles. Future research in AM may involve the design of new materials suitable for AM, which requires desirable grain structure that can be achieved directly by the AM process or with the aid of post-processing heat treatment. The new materials for AM also need to have good compatibility to AM thermal histories, such as low susceptibility to hot cracking.
